# The Ten-Point Treatment Programme: Design and Evaluation of an Easy Read Document in a Forensic Learning Disability Unit

**DOI:** 10.1192/bjo.2024.91

**Published:** 2024-08-01

**Authors:** Ayomipo Amiola, Holly Anna Marler, Carly Weeks, Vanessa Barnes, Regi Alexander

**Affiliations:** 1 Hertfordshire Partnership University NHS Foundation Trust, Norwich, United Kingdom; 2 University of Hertfordshire, Hatfield, United Kingdom

## Abstract

**Aims:**

There has been criticism surrounding the lack of clarity regarding treatments offered within forensic inpatient units for people with learning disability and co-existing mental health problems. The Ten-Point Treatment Programme is a framework for treatments within such settings. It incorporates the four stages of assessment and motivational work, foundation and offence-specific treatments, consolidation and relapse prevention and finally discharge management. Although evidence based and evaluated in outcome studies, explaining its content to those with learning disability can be problematic. Communication difficulties affect the way information is comprehended and interpreted from both a linguistic and pragmatic perspective in this group. The provision of Easy Read information can address this difficulty.

Our aim was to co-produce, with experts by experience, an easy read version of the Ten Point Treatment Programme; and to evaluate this resource.

**Methods:**

This was a quality improvement project within an in-patient medium secure unit in England. The co-production of the easy read version was led by two speech and language therapists, two psychiatrists, one Education Manager and two experts by experience. The latter advised on content, wording, format and font. Content was adapted in line with standard easy read requirements and guidelines. Following a focus group meetings, a provisional easy read version was approved and introduced in the service. This service innovation was evaluated through semi-structured interviews with six experts by experience and ten multidisciplinary team members who had used the resource. Responses were transcribed and subjected to thematic analysis.

**Results:**

The three main themes covered in the evaluation responses related to accessibility, appearance and usefulness. The sub-themes under accessibility were the simplicity of vocabulary and short sentence length. Regarding appearance, the key sub-themes were about the effective use of colour, the inclusion of relevant and meaningful images, and the balance between words and pictures. On usefulness, the main sub-theme was about understanding the treatment pathway better and hence feeling motivated to engage. This was reflected by the staff group as well. There were some comments on accessibility that were less positive, including service user indications that the number and complexity of words were still high.

**Conclusion:**

The co-produced easy read version of the Ten-point treatment programme has been received positively by service users and staff. For both groups, it brings clarity about the treatment pathway and its stages. It is incorporated into the admission pack for new admissions and features in new staff induction programmes.

